# Discovery of Novel Multi-target Inhibitor of angiotensin type 1 receptor and neprilysin inhibitors from Traditional Chinese Medicine

**DOI:** 10.1038/s41598-019-52309-z

**Published:** 2019-11-07

**Authors:** Xiaoqian Huo, Liansheng Qiao, Yankun Chen, Xi Chen, Yusu He, Yanling Zhang

**Affiliations:** School of Chinese Materia Medica, Beijing University of Chinese Medicine, Beijing, 100102 China

**Keywords:** Virtual screening, Receptor pharmacology

## Abstract

Angiotensin II type-1 receptor–neprilysin inhibitor (ARNi) is consisted of Angiotensin II type-1 receptor (AT1) antagonist and neprilysin (NEP) inhibitor, which could simultaneously increase the vasodilators of the natriuretic peptides and antagonize vasoconstrictors of Ang II. ARNi has been proved a superior effect and lower risks of death on chronic heart failure (CHF) and hypertension. In this paper, ARNi from Traditional Chinese Medicines (TCM) was discovered based on target combination of AT1 and NEP by virtual screening, biological assay and molecular dynamics (MD) simulations. Two customized strategies of combinatorial virtual screening were implemented to discover AT1 antagonist and NEP inhibitor based on pharmacophore modeling and docking computation respectively. Gyrophoric acid (PubChem CID: 135728) from *Parmelia saxatilis* was selected as AT1 antagonist and assayed with IC_50_ of 29.76 μM by calcium influx assay. And 3,5,3′-triiodothyronine (PubChem CID: 861) from *Bos taurus do*mesticus was screened as NEP inhibitor and has a dose dependent inhibitory activity by biochemistry fluorescence assay. Combined with MD simulations, these compounds can generate interaction with the target, key interactive residues of ARG167, TRP84, and VAL108 in AT1, and HIS711 in NEP were also identified respectively. This study designs the combinatorial strategy to discover novel frames of ARNi from TCM, and gyrophoric acid and 3,5,3′-triiodothyronine could provide the clues and revelations of drug design and therapeutic method of CHF and hypertension for TCM clinical applications.

## Introduction

Chronic heart failure (CHF) is the progressive inability of the heart to supply inadequate blood flow to vital organs. Although diagnosis and treatment have been developed significantly, CHF caused by cardiovascular diseases (CVD) and hypertension remains a severe damage to human health. The ventricular remodeling plays an important role in the developing of CHF. However, despite the use of angiotensin-converting enzyme (ACE) inhibitors, β-blockers, and mineralocorticoid receptor, antagonists patients remain at high risk of worsening heart failure^1^. Luckily, angiotensin II type 1 receptor-neprilysin inhibitors, which consist of the angiotensin II receptor type 1 (AT1) inhibitors and beta blockers, provide new methods to the therapy of CHF^2^.

NEP, known as neprilysin, is a zinc-dependent metalloprotease, which is encoded by the MME gene and expressed in a wide variety of tissues^3^. The key biological activity of NEP is hydrolysis of the cardiovascular peptides^4^. The inhibition of neprilysin provided the benefits like vasoactive substances and exerts favorable effects to the patients with heart failure, when combined the agents acted on detrimental neurohormonal systems. However, the inhibitor of neprilysin also activates the renin-angiotensin system, possibly cause angiotensin increase^5^. The combined use of a neprilysin inhibitor with angiotensin receptor blocker is a method for better effect with safety^6^.

Researches have proved that combination inhibition of AT1 and NEP could achieve superior effect and lower risks of death than inhibition of single target^7,8^. LCZ696, consisted of neprilysin (NEP) inhibitors (sacubitril, PubChem CID:9811834) and angiotensin II receptor type 1 (AT1) antagonists (valsartan), is developed to treat CHF and hypertension^7^. Furthermore, LCZ696 was the first drug which outperformed the ACE inhibitor (enalapril, PubChem CID:5388962) for treating CHF in head to head comparative trial^8^. Though the single target drugs have a common use and effect in clinic, the multi-target formulas also have significantly meaning in the drug discovery. Further development of angiotensin type 1 receptor-neprilysin inhibitors (ARNi) is essential to treat CHF with fewer side effects.

Treatment effects of Traditional Chinese Medicine (TCM) for CHF and hypertension have special advantages, including multi-component, multi-target and multi-pathway^9^. For example, research indicated that Qili Qiangxin capsules had good effect for CHF and could reduce the levels of N-terminal pro-B-type natriuretic peptide (NT-proBNP)^10^. And some of the TCM herbs and formulas also have been proven to have direct or indirect regulatory effects of AT1 and NEP. The [6]-Gingerol derived from *Zingiber officinale* was identified as a direct AT1 antagonist^11^. The TCM formulas of Xiaxi oral liquid and Yiqi Huaju formula were also proven to down-regulate the transcription and translation of AT1 gene^12,13^. Meanwhile, some of flavonoids derived from *Epilobium angustifolium* had been reported as direct NEP inhibitors, including oenothein B and Quercetin-3-O-glucuronide^14^. The aqueous extract of *Uncaaria rhynchophylia* also has direct inhibitory activity of NEP^15^. Although target combination of AT1 and NEP is critical for treating CHF and hypertension, ARNi has never been discovered from TCM. Thus, it is significant to discover ARNi from TCM for CHF.

In this paper, combinatorial virtual screening of ligand-based and structure-based approaches was utilized to discover TCM ARNi. Pharmacophore and docking models of AT1 antagonists and NEP inhibitors were constructed firstly for the screening of TCM compounds. Two screening processes of NEP inhibitors and AT1 antagonists were implemented to discover potential TCM ARNi. Molecular dynamics simulation was further utilized to prove the interaction of TCM ARNi. Finally, biological assays were utilized to identify the interaction of targets and ARNi. This study provided the novel framework of efficient screening of ARNi leading compounds from TCM for further drug design and development. And the TCM combination of ARNi was also beneficial discovery of original ARNi of TCM for treating CHF and hypertension.

## Materials and Methods

### Ligand-based pharmacophore modeling of ARNi

The 3D quantitative pharmacophore hypotheses of AT1 antagonists and NEP inhibitors were constructed respectively by 3D QSAR Pharmacophore Generation (HypoGen) within Discovery Studio 4.0 (DS). Three-dimensional structures of all compounds in training set and test set were constructed and full minimized in CHARMm force field with MMFF94 partial charge. And 255 conformations of compounds were created for pharmacophore modeling within the relative energy threshold of 20.0 kcal/mol by BEST model.

The universal set of 120 active AT1 antagonists was derived from the Binding Database (http://www.bindingdb.org/bind/index.jsp). Wherein, 20 compounds with structural diversity were selected as the training set, and the range of IC_50_ in training set was across 8 orders of magnitude (Fig. S1). Then 300 inactive compounds were selected randomly from Drugbank without AT1 antagonistic activity. According to the feature analysis of training set, features of hydrogen bond acceptor (A), hydrophobic (H), ring aromatic (R) and hydrogen bond donor (D) and ionizable negative (N) were identified for modeling.

The universal set of 46 human NEP inhibitors was also obtained from the Binding Database. A set of 23 compounds was rationally selected as the training set based on the activity value range (5 orders of magnitude) and structural flexibility of compounds. Additionally, the remaining 23 compounds were regarded as test set with 69 inactive compounds. Chemical information of the training set of NEP pharmacophore was shown in Fig. S2. According to the feature mapping analysis, pharmacophore features of R, H, A, D and N were selected during the pharmacophore generation.

During the process of pharmacophore modeling, an activity uncertainty of three was defined for each compound, and the maximum excluded volumes (Ev) value was set to 5. In order to validate the accuracy of pharmacophore models, multiple internal and external indexes were utilized to describe the screening efficiency of pharmacophore. According to HypoGen algorithm, the correlation coefficient of training set and cost function were calculated as internal evaluation indexes for pharmacophore. Also, the test set was utilized to compute external indexes, including hit rate of active compounds (*HRA*), identify effective index (*IEI*) and comprehensive appraisal index (*CAI*)^16,17^. Then, by considering all these indexes, the best pharmacophore model was selected as a query for virtual screening of TCM ARNi with flexible fitting method.

### Docking modeling of ARNi

A classic docking algorithm of CDOCKER was utilized for docking studies of ARNi. CDOCKER uses a CHARMm-based molecular dynamics scheme to dock ligands into a receptor binding site and generate the active binding poses of lead ligands. In this study, the crystal structures of AT1 (PDB ID: 4YAY) and NEP (PDB ID: 2QPJ) from the RCSB Protein Data Bank (http://www.rcsb.org) were utilized to construct docking models by CDOCKER. The crystal structures were prepared which included removing water, adding hydrogen atoms and building loops for the construction of models. Then, the binding site was determined by initial ligand within crystal structure. Furthermore, initial ligands of ZD7155 and phosphoramidon were re-docked into 4YAY and 2QPJ for computing root mean square deviation (RMSD) between the pose of docking results and origin pose. RMSD used as a quantitative measure by measuring the average distance between the heavy atoms in the molecules, and evaluating the accuracy of docking algorithm, respectively. In general, RMSD less than 2.00 Å indicated that a docking algorithm would reproduce the experimentally observed binding mode. Besides, valsartan and sacubitril were docked into respective binding site, and the poses of the docked ligands were analyzed to obtain the key residues. TCM ARNi hit by pharmacophore model were further filtered by docking models.

### Structure-based pharmacophore modeling of AT1 agonists

A crystal structure of AT1 complexes with inverse agonists (PDB ID: 4ZUD) was employed to generate structure-based pharmacophore (SBP) model^18^. Preparation of AT1 and definition of binding site were implemented as the method of docking modeling. Primary features, including A, D and H feature, were displayed in all the possible interactive points available at the binding site. Then, neighboring primary features within 2.00 Å were clustered to the same features, and multiple SBP models were generated by random combination of 4 to 8 pharmacological features from the clustering results^19^. Ev features were added based on residues in binding site. In order to evaluate the SBP models, testing set for SBP was constructed with the same structure framework including three AT1 agonists, three AT1 inverse agonists, and three AT1 antagonists (Fig. S3)^20,21^. A model which could distinguish between agonists and antagonists was selected as the optimal SBP model to eliminate the effect of AT1 agonists in screening process.

### Calcium influx assay of AT1 antagonists

Calcium influx assay of AT1 antagonists was implemented by Molecular Devices fluorescent imaging plate reader (FLIPR, Shanghai, China). Stable cell lines HEK293/Ga15/AT1 and Fluo-8 Calcium Assay Kit were provided by HD Biosciences (Shanghai, China). AT1 agonist of angiotensin II and AT1 antagonist of telmisartan were purchased from Sigma-Aldrich (St. Louis, MO, USA). TCM AT1 antagonist of gyrophoric acid was purchased from Shanghai Yuanye Bio-Technology Co., Ltd. (Shanghai, China).

The HEK293/Ga15/AT1 cells have a high expression of AT1, and were used to verify the activity of AT1 agonist. HEK293/Ga15/AT1 cells were seeded at a density of 1.5 × 10^4^ cells/well in 384-well assay plate coated with matrigel (BD), and were cultured 16–24 hours in complete media^11^. In the following day, supernatant was replaced with Fluo-8 Loading Dye, and the plate was incubated at 37 °C in dark for 1 hour. Then, increasing concentrations of telmisartan and gyrophoric acid were added into cells, which continued to be incubated at room temperature in the dark for 15 minutes. The intracellular calcium influx was assayed by monitoring Fluo-8 fluorescence in all wells simultaneously (λ_ex_/λ_em_ = 470–495 nm/515–575 nm). A 10 seconds baseline was performed, and the FLIPR added positive (1 µM angiotensin II) control to each well and fluorescence intensity was captured every 1 s for 100 s. Dose-response curves and IC_50_ values were determined using GraphPad Prism 5 software.

### Biochemistry fluorescence assay of NEP inhibitors

Biochemistry fluorescence assay of NEP inhibitors were performed by Molecular Devices Flexstation III (Molecular Devices) which can provided the fluorescence detection^22^. NEP can catalyze the substrate into fluorescent production. So the fluorescence intensity can indicate the inhibition activity of compounds. When the compounds with inhibition activity put into the system, the enzyme produce less production and the fluorescence would weaken. Recombinant human NEP expressed and purified from HEK 293 cells and positive NEP inhibitor of DL-Thiorphan was supplied by Sigma-Aldrich. Substrate of DNS-Gly-(pNO_2_)Phe-β-Ala (DGNPA) for NEP were chemically synthesized by the Beijing Scilight Biotechnology Ltd., Co. (Beijing, China). TCM NEP inhibitor of 3,5,3′-triiodothyronine was purchased from Shanghai Yuanye Bio-Technology Co., Ltd.

The sample divided into six groups. Group 1 was considered 100% hydrolyze group, with substrate and enzyme. Group 2 added substrate without the enzyme, and considering as control group. Group 3 was received the substrate with enzyme and DL-Thiorphan in 50 μM as the positive control. The control group was used to verify the experiment system. Group 4, 5, 6 was given the substrate with enzyme and 3,5,3′-triiodothyronine at the doses of 50, 100, and 200 nM.

NEP (final concentration 1 µg/mL) was incubated for 15 min at 37 °C in a total volume of 100 µL of 50 mM Tris/HCl buffer, pH 7.4 (final concentration 1 μM) and increasing concentrations of 3,5,3′-triiodothyronine^23,24^. The reaction was initiated by the addition of the DGNPA to a final concentration of 37 µM, and was stopped after 30 min at 37 °C by adding 100 µL of DMSO. The 200 µL mixture was measured by fluorescence (λ_ex_ = 342 nm, λ_em_ = 525 nm). All the measurements were done in black 96-well plates. Mixtures of 0% hydrolysis consisted of the buffer and DGNPA only, while mixtures of 100% hydrolysis were the group without inhibitor^25^. The inhibition ratio of NEP was computed by reference to 100% hydrolysis and the inhibition activity values of 3,5,3′-triiodothyronine were determined accordingly.

### Molecular dynamics simulation of ARNi

Four atomistic molecular models were constructed using docking results of AT1-valsartan, AT1-gyrophoric acid, NEP-sacubitril, and NEP-3,5,3′-triiodothyronine. Then, MD simulations were further implemented for these four models with GROMACS 5.0.2. The topology files of ligands were generated by the GlycoBioChem PRODRG2 Server (http://davapc1.bioch.dundee.ac.uk/cgi-bin/prodrg)^26^. The complex systems were solvated by explicit SPC water model in cubic boxes maintaining a minimum 12 Å distance from cube edge.

For the MD of AT1 atomistic models, the protein was embedded within a hydrated dipalmitoylphosphatidylcholine (DPPC) bilayer, which was obtained from Tieleman Web page^27,28^. The simulation cell consisted of ~91400 atoms using the GROMOS96 53a6_lipid.ff force field. Nine chloride ions were added by randomly replacing water molecules to achieve a neutral simulation cell. Each complex system was then minimized using a steepest descent integrator with 5000 steps. A 500 ps NVT equilibration was performed at 300 K with position restraints applied to protein and ligand in order to relieve any bad contacts at the residues solvent interface. Then a 5000 ps NPT simulation was conducted, and pressure was coupled to 1.0 atm using the Parrinello-Rahman method. Then, for the simulation of NEP complex systems, the simulation cell consisted of ~148900 atoms using the GROMOS96 43a1 force field. Nine sodium ions were added by randomly replacing water molecules to achieve a neutral simulation cell. A 500 ps NVT equilibration was performed for temperature control and a 1000 ps NPT ensemble at 1.0 bar along with constant pressure.

Upon the two phases of equilibration, the 20 ns MD was performed and the binding stability of the four complexes was analyzed. The MD trajectory was determined for RMSD of protein backbone and total energy. At the same time, RMSF and H-bond occupancy rates were also analyzed to identify the key interactive residues of complex systems.

## Results and Discussion

### Virtual screening for AT1 antagonists from TCM

**Figure 1:**
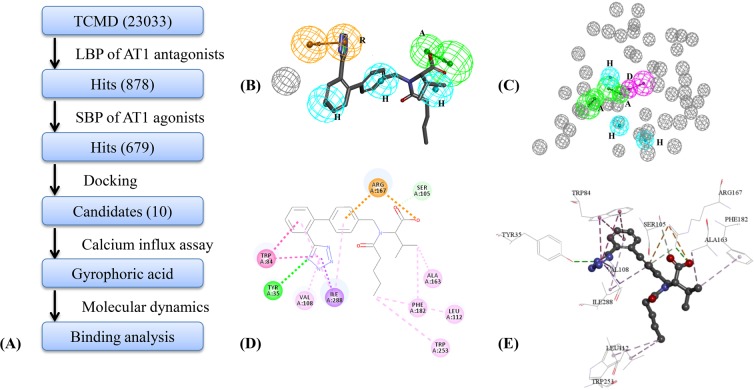
Virtual screening of AT1 antagonists. (**A**) Work-flow of virtual screening to discover AT1 antagonists. (**B**) Optimal LBP model of AT1 antagonists mapping with valsartan. (**C**) Optimal SBP model of AT1 agonists. (**D**) The 2D docking results between AT1 and valsartan. (**E**) The 3D docking results between AT1 and valsartan. (Green circles represent the hydrogen bond accept (**A**). Yellow circles represent aromatic ring aromatic interaction (**R**). Circles in cerulean blue represent the hydrophobic interaction (**H**)).

In order to screen AT1 antagonists from TCM, a work-flow of molecular modeling and virtual screening was implemented and shown in Fig. 1A ^29,30^. Ligand-based pharmacophore (LBP) of AT1 antagonists was first constructed to screen Traditional Chinese Medicine Database (TCMD). As a typical GPCR, agonists and antagonists of AT1 act on the same binding site, so SBP of AT1 agonists were further constructed for eliminating the negative effect on AT1 agonists. Then, hit compounds obtained by pharmacophore were docked into docking model of AT1 antagonists, and top 10 compounds were retained as potential candidates. The AT1 antagonistic activity of candidate was evaluated by calcium influx assay, and the binding interaction of AT1 antagonists was analyzed by MD.

Ten pharmacophore hypotheses of AT1 antagonists were generated by HypoGen algorithm (Table 1). In HypoGen, cost function was an internal index to indicate the significance of models. Null Cost of ten hypotheses was 228.61, and △cost was difference value between Null Cost minus Total Cost of each hypothesis. The △cost value of ten hypotheses greater than 60 indicated that there is generally a high probability and low contingency of models^31^. From the Table 1, correlation values between predicted and experimental activity of training set for pharmacophore models were greater than 0.8, which indicated pharmacophore models had better ability to predict activity of compounds^31^. Then testing set was used to compute external indexes of the pharmacophore models in order to analyze the capabilities of identifying active compounds from non-active compounds and distinguishing the higher activity compounds from lower activity compounds. Three external indexes were computed, including *HRA*, *IEI*, and *CAI*. The hypo 7 has a satisfied CAI, an acceptable correlation and △cost. According to *CAI* and correlation, Hypo7 was regarded as the best pharmacophore (Fig. 1B) with one A feature, three H features, one R feature and one Ev. Wherein, valsartan could be mapped with Hypo7 and the fit value was 0.88. According to the screening of TCMD, 878 TCM compounds were obtained to further screen for AT1 antagonists.

**Table 1:** The evaluation results of pharmacophore models.

Hypo	Features	*HRA%*	*IEI*	*CAI*	Correlation	Total cost	Δcost
1	AHHHR Ev2	70	3.59	2.51	0.90	133.25	95.37
2	AAH	100	1.56	1.56	0.95	135.52	93.09
3	AAHHH Ev2	98	2.47	2.42	0.86	137.34	91.27
4	AAH Ev2	100	1.55	1.55	0.95	139.22	89.40
5	AAH Ev2	100	1.53	1.53	0.95	140.00	88.62
6	AAH Ev2	100	1.52	1.52	0.92	141.47	87.14
**7**	**AHHHR Ev1**	**98**	**3**.**02**	**2**.**96**	**0**.**83**	**141**.**49**	**87**.**13**
8	AAR Ev3	100	1.40	1.40	0.93	142.29	86.32
9	AHHH Ev3	99	1.69	1.68	0.88	144.69	83.92
10	AHHHR Ev2	74	3.40	2.52	0.86	144.80	83.81
11	AAHH Ev2	100	1.23	1.23	0.81	165.39	102.64
12	AAHH Ev1	100	1.30	1.30	0.86	146.05	121.98
13	ADHH Ev5	43	1.74	0.76	0.84	157.37	110.67
14	AHHN Ev4	96	4.00	3.83	0.82	154.57	113.47
15	AAR Ev3	100	1.28	1.28	0.87	146.25	121.79
16	AAHH Ev5	96	1.29	1.24	0.87	142.87	125.17
17	ADHH Ev5	96	4.00	3.83	0.84	157.37	110.67
18	AHHN Ev4	43	1.48	0.64	0.82	154.57	113.47
19	AAR Ev3	100	1.28	1.28	0.87	146.25	121.79
**20**	**DHNR Ev3**	**83**	**4.00**	**3.30**	**0.87**	**137.53**	**130.51**

SBP model of AT1 agonists was constructed by 4ZUD. The 340 pharmacophore features were identified in binding site of 4ZUD with the a sphere radius of 8.14 Å. A set of 28 primary features was obtained based on clustering of features, including nine D features, eight A features, and eleven H features. The testing set was utilized to evaluate the screening efficiency of SBP models obtained by random combination of 4 to 8 primary features. The optimal SBP model was identified, which could match AT1 agonists with fit value greater than 0.5 and match other compounds with fit value less than 0.5 (Table S1). As a result, the optimal SBP model consisted of six features, including two A features, one D feature, and three H features (Fig. 1C). The optimal SBP model was utilized to screen and eliminate 199 AT1 agonists from screening results of LBP.

Because structure of AT1 has been revealed by Zhang^32^, screening mode of AT1 antagonists would produce subversive change against previous homology modeling method^33^. The binding pocket of 4YAY was created with a sphere radius of 8.82 Å around the initial compound (ZD7155). The RMSD value between the re-docked and original conformations of ZD7155 was 0.49 Å (< 2.00 Å), which indicated the reliability of docking model^34^. Approved AT1 antagonist of valsartan was also docked into binding site for analyzing the key interactive residues. Two and three dimensional interaction results of valsartan were shown in Fig. 1D,E. The CDOCKER ENERGY of valsartan was −32.52 kcal/mol and binding free energy was −251.56 kcal/mol. Finally, docking model was used to screen hits from pharmacophore models and top 10 potential AT1 antagonists were obtain from TCM (Table S2). Wherein, gyrophoric acid was regarded as the potential AT1 antagonists, which was available and utilized for further biological assay.

### Virtual screening for NEP inhibitors from TCM

**Figure 2:**
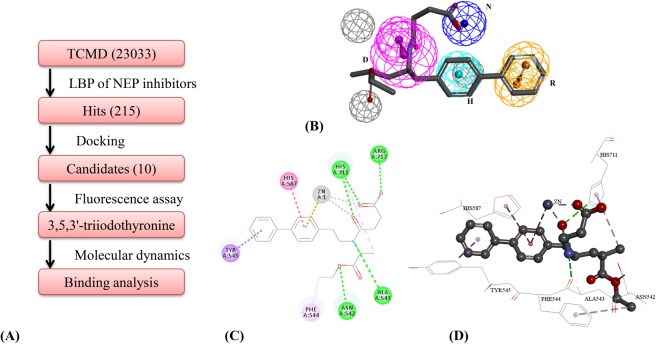
Virtual screening of NEP inhibitors. (**A**) A work-flow of virtual screening to discover NEP inhibitors. (**B**) Matching graph of optimal LBP of NEP inhibitors and sacubitril. (**C**) 2D interaction between NEP and sacubitril. (**D**) 3D interaction between NEP and sacubitril. (Pink circles and pink dot-dashed line represent hydrogen bond donor (**D**). Yellow circles represent aromatic ring aromatic interaction (R). Circles in cerulean blue represent the hydrophobic interaction (H). Blue circle represent ionizable negative (N). Green dot-dashed line represent hydrogen bond acceptor(**A**)) .

A work-flow of molecular modeling and biological assays was also implemented to screen and validate NEP inhibitor (Fig. 2A). The optimal LBP model was first generated and utilized to screen potential NEP inhibitor from TCMD. Then, molecular docking was employed to refine the hits obtained from pharmacophore-based screening and obtain potential NEP inhibitor. The NEP inhibitory activity of potential candidate was evaluated by biochemistry fluorescence assay. And the binding mode of NEP-ligand was analyzed by docking results and MD simulation.

According to HypoGen algorithm, top 10 pharmacophore hypotheses of NEP inhibitors were also constructed and detailed in Table 1. Multiple internal and external indexes were used to validate these pharmacophore models. Null cost of 10 hypotheses was 268.04 in cost analysis. Values of △cost of each hypothesis were greater than 60 and the correlation coefficient values of 10 hypotheses also were greater than 0.8, which indicated the reliability of pharmacophore. According to testing set, three external indexes of *HRA*, *IEI*, and *CAI* were also computed to evaluate pharmacophore. Having comprehensively appraised *CAI* and correlation, Hypo20 was chosen as the best pharmacophore model (Fig. 2B). Hypo20 consisted of four features, including one N feature, one D feature, one H feature, one R feature, and three Ev features. The hypo 20 has a quality correlation and cost. The correlation, *HRA*, *IEI*, and *CAI* were 0.87, 83%, 4.00, and 3.30, respectively. Then, Hypo20 was used as a template to screen the TCMD. By using Hypo20, a hit list of 215 compounds with drug-like property was obtained from TCMD.

As the zinc-dependent metalloprotease, structure of NEP was revealed by Oefner^35^. According to CDOCKER algorithm, molecular docking model of NEP inhibitors was constructed, and the binding site around the initial compound (phosphoramidon) was created with a sphere radius of 10.81 Å. The RMSD value between the re-docked and original conformations of phosphoramidon was 1.38 Å, which indicated the reliable docking model. And approved NEP inhibitor of sacubitril was docked into binding site. The CDOCKER ENERGY of sacubitril was 57.56 kcal/mol and binding free energy was −111.91 kcal/mol (Fig. 2C,D). Then, 215 TCM compounds were docked into the binding site, and the top 10 candidates with high-scoring function were retained for further biological evaluation (Table S3). Among the top 10 compounds, 3,5,3′-triiodothyronine is a potential lead candidate as NEP inhibitor, which had a higher estimated activity based on the combinatorial screening.

### Biological evaluation for TCM ARNi

**Figure 3:**
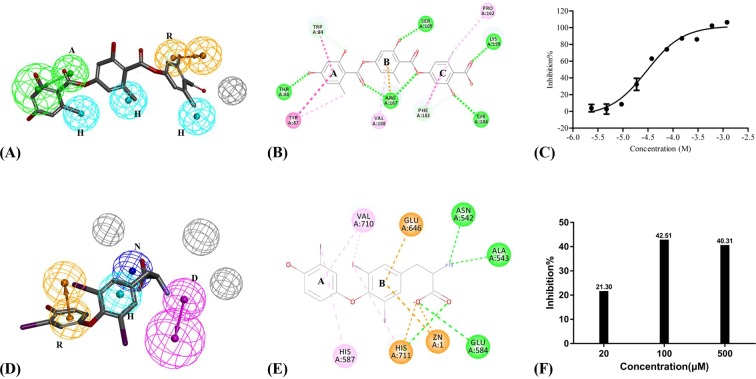
Screening and assay results of TCM ARNi. (**A**) Matching graph of LBP model of AT1 antagonists and gyrophoric acid. (**B**) Docking result between AT1 and gyrophoric acid. (**C**) Dose-response curves for gyrophoric acid using calcium influx assay in HEK293/Ga15/AT1. (**D**) Matching graph of LBP model of NEP inhibitors and 3,5,3′-triiodothyronine. (**E**) Docking result between NEP and 3,5,3′-triiodothyronine (**F**) Dose-response column diagram for 3,5,3′-triiodothyronine by fluorescence assay.

According to the pharmacophore matching, fit value of gyrophoric acid was 0.76 (Fig. 3A). The CDOCKER ENERGY of gyrophoric acid was −26.26 kcal/mol and binding free energy was −42.35 kcal/mol based on docking result. The RMSD between pharmacophore- and docking-based conformations of gyrophoric acid was 2.13 Å, which reflected the consistency of two models. Carbonyl oxygen in A-ring (Fig. 3B) of gyrophoric acid could form hydrogen bond with ARG167 and also map with A feature in pharmacophore. Methyl group in A-ring could perform hydrophobic effect with TYR87 and map with H feature in pharmacophore. The C-ring of gyrophoric acid could map with R feature with PHE182, and methyl group in C-ring could produce hydrophobic effect with PHE182 and PRO162 matched with H feature. Gyrophoric acid from virtual screening was further assessed to antagonize AT1 in recombinant cells (HEK293/Ga15/AT1 cells) using the intracellular calcium influx assay. Telmisartan was used as positive control for AT1 assays with IC_50_ of 1.83 nM, which were very close to literature^36,37^. Gyrophoric acid was confirmed to be a validated AT1 antagonist with IC_50_ of 29.76 μM (Fig. 3C).

The fit value of 3,5,3′-triiodothyronine was 0.80 matched with pharmacophore of NEP inhibitors (Fig. 3D). According to docking of 3,5,3′-triiodothyronine, the CDOCKER ENERGY was −69.74 kcal/mol and binding free energy was −161.10 kcal/mol. The RMSD between pharmacophore and docking-based conformations of 3,5,3′-triiodothyronine was 1.77 Å, which indicated the similarity and reliability of two models. The carboxyl group in 3,5,3′-triiodothyronine could form electrostatic interaction with HIS711 and Zn^2+^ which confirmed using the N feature in pharmacophore model (Fig. 3E). Additionally, A-ring of 3,5,3′-triiodothyronine has formed a hydrophobic interaction with VAL710, which further confirmed using the R feature in the pharmacophore model. The amino group was mapped with D feature and formed hydrogen bond interactions with ASN542 and ALA543. The biochemistry fluorescence assay was utilized to estimate the screening results of NEP. The control group has 85% inhibition rate in the NEP assays, which verify the rationality experiment system. Then, 3,5,3′-triiodothyronine has obvious dose-dependent inhibitory activity of NEP. The results implied the observed inhibitory effects of 3,5,3′-triiodothyronine. And it maybe a potential NEP inhibition for further studies.

### Binding interaction analysis of TCM ARNi

The key binding interactions were analyzed based on docking results of AT1 antagonists firstly. The hydrogen bond interactions were produced with TYR35 and ARG167 between AT1 and initial ligand (ZD7155). And the special Pi-Pi stack of TRP84 and pyridine in ZD7155 was also analyzed related to the activity. These three critical interactions have been proved by Zhang^32^. Besides, a set of ILE31, PHE77, VAL108, ALA163, PRO285, ILE288, and TYR292 could form hydrophobic interactions with AT1 (Table S4). And valsartan could form hydrogen bond interactions with ARG167 and TYR35, which was also analyzed by ZD7155 and verified the key residue of AT1 antagonists. The hydrophobic interactions between valsartan and TRP84, VAL108, ALA163, and ILE288 were also found the same as ZD7155. Compared with the interactions of gyrophoric acid, hydrogen bond residue of ARG167, and hydrophobic residues of TRP84 and VAL108 were regarded as the key residues for AT1 antagonists.

**Figure 4:**
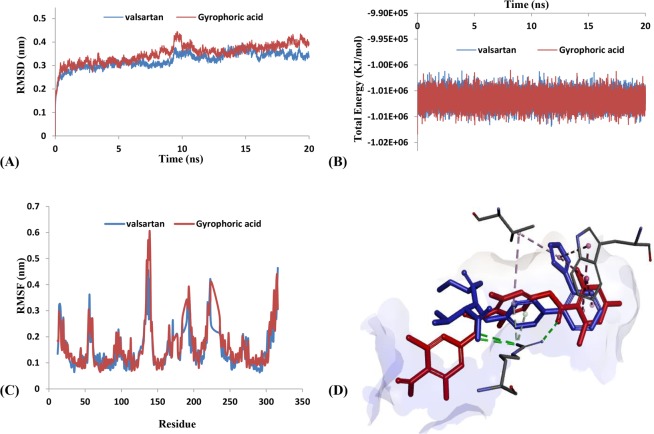
Binding analysis of AT1 antagonists. (**A**) RMSD of AT1-ligands complexes. (**B**) Total energy of AT1-ligands complexes. (**C**) RMSF corresponds to MD trajectory of AT1. (**D**) 3D key interaction of valsartan (blue) and gyrophoric acid (red).

Molecular dynamics simulation was further implemented to verify the interactive residues of AT1 antagonists. The RMSD trajectories and total energy profiles of AT1-valsartan and AT1-gyrophoric acid complexes are shown in Fig. 4A,B, respectively. The RMSD and total energy of two systems had similar trend and were stabilized within 20 ns. Then, root mean square fluctuation (RMSF) (Fig. 4C) and H-bond occupancy rates were utilized to analyze key residues obtained from docking computation^38,39^. Gyrophoric acid displayed a similar variation of flexibility of residues compared to valsartan based on RMSF. Actually, ARG167, TRP84, and VAL108 had similar and low fluctuation in two systems. Valsartan could form H-bond at ARG167, and the occupancy rate could reach as 62.50%. And gyrophoric acid formed H-bonds at ARG167, with an high occupancy rate of 87.35%. It indicated these three residues of ARG167, TRP84, and VAL108 were important to AT1 antagonists (Fig. 4D).

**Figure 5:**
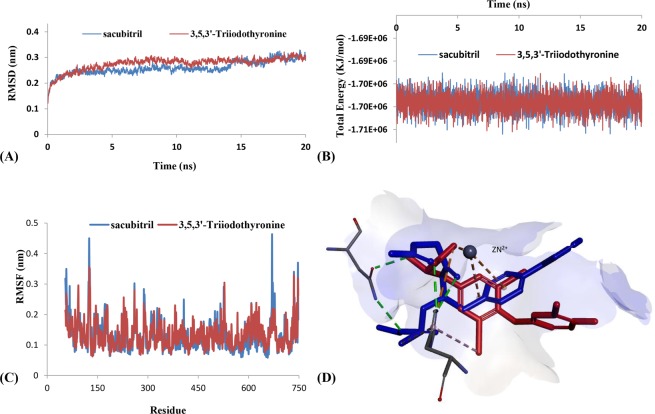
Binding analyses of NEP inhibitors. (**A**) RMSD of NEP-ligands complexes. (**B**) Total energy of NEP-ligands complexes. (**C**) RMSF corresponds to MD trajectory of NEP. (**D**) 3D key interaction of sacubitril (blue) and 3,5,3′-triiodothyronine (red).

The 3D binding poses of initial ligand (phosphoramidon) and sacubitril were also analyzed for interactive residues (Table S5). Both two NEP inhibitors could form hydrogen bond interactions with ASN542 and HIS711 and strong electrostatic interactions at the active site with Zn^2+^, which were consistent with Misawa^40^. These three key interactions were also formed between 3,5,3′-triiodothyronine and NEP. MD simulation of NEP-sacubitril and NEP-3,5,3′-triiodothyronine were run to verify the key residues from docking poses. The RMSD and total energy were analyzed to evaluate the stability of two systems. The RMSD trajectories (Fig. 5A) and total energy (Fig. 5B) of the two complexes reached well equilibrated within 20 ns. In addition, RMSF values for the two complexes were further calculated to evaluate the flexibility of the residues. The results showed that 3,5,3′-triiodothyronine displayed similar total energy and residue variation to sacubitril. According to the analysis of fluctuation score at residues, ASN542 and HIS711 exhibited the similar and low fluctuation between 3,5,3′-triiodothyronine and sacubitril. However, ASN542 could not form stable hydrogen bond interaction in both two compounds during MD process. Sacubitril formed H-bonds at HIS711 (25.19%), and 3,5,3′-triiodothyronine formed H-bonds with HIS711 (76.22%). Actually, HIS711 was an important residue because of forming not only hydrogen bond interaction, but also hydrophobic interaction in two compounds.

In this study, gyrophoric acid from *Parmelia saxatilis* and 3,5,3′-triiodothyronine from *Bos taurus domesticus* were identified as the AT1 antagonist and NEP inhibitor, respectively. Previous researches have shown gyrophoric acid was regarded as the protein tyrosine phosphatase 1B (PTP1B) inhibitor for treatment of diabetes^41^. Meanwhile, gyrophoric acid has promising activity of antioxidant^42^ and lower anti-proliferation activity than other lichen secondary metabolites^43^. As the result of our research, gyrophoric acid was a natural AT1 antagonist, which could be used as the important component of ARNi for CHF and hypertension.

Meanwhile, 3,5,3′-triiodothyronine is not only a physiological activity constituent in goitre of human, but also the chemical component from goitre of animal TCM. The parent nucleus of 3,5,3′-triiodothyronine has a reaction with the structure of NEP, which may be a potential leading compounds for further compounds design. The antihypertensive effect of 3,5,3′-triiodothyronine has been verified in the hypothyroidism. The screening of NEP inhibitor has indicated the interaction between NEP and 3,5,3′-triiodothyronine, which may be a possible mechanism. Then the activity assays had verified the inhibitory activity of 3,5,3′-triiodothyronine. The inhibition suggests that 3,5,3′-triiodothyronine may be a potential leading compounds for the original ANRi development.

3,5,3′-triiodothyronine was also found as a natural NEP inhibitor in present study, which could provide novel formula component for ARNi. Actually, AT1 and NEP belong to different protein superfamily. According to similarity analysis of binding sites, the TM-score between AT1 and NEP was 0.21 by TM-align analysis^44^, which indicated two targets have low structural similarity. Therefore, the formula drug is a better method to regulate these two targets at the same time. TCM is commonly used as the formula to treat diseases with the long history and numerous clinical data, which could provide the clue to discover novel formula of ARNi.

## Conclusion

In this study, a method included two work flows of virtual screening and biological assays were utilized to discover ARNi from Chinese herbs. Potential AT1 antagonist and NEP inhibitor were identified based on combinatorial screening of LBP, SBP and molecular docking, and biological assay were used to verify results. Gyrophoric acid from *Parmelia saxatilis* was investigated for AT1 antagonistic activity with IC_50_ of 29.76 μM. Demonstrated by MD simulation, gyrophoric acid could form key interactive residues with AT1, including ARG167, TRP84, and VAL108. Then, 3,5,3′-triiodothyronine from *Bos taurus domesticus* has obvious inhibitory activity of NEP. HIS711 and Zn^2+^ were regarded as the key interactive features for NEP inhibition.

According to the combination of virtual screening and biological assay, TCM ARNi was identified, including gyrophoric acid for AT1 antagonism and 3,5,3′-triiodothyronine for NEP inhibition. This ARNi could provide the novel chemical frame and design idea for ARNi formula. Actually, the systematic mechanism of this ARNi should also be further investigated and verified by cell and animal experiments, especially the off-target effect and dose proportion. In conclusion, this paper provided lead compounds of ARNi from TCM, which indicated it is necessary to discover the novel clue for formula design from traditional medicine.

## Supplementary information

Table S1, Table S2, Table S3, Table S4, Table S5, Figure S1, Figure S2, Figure S3, Table S6, Table S7
