# Supplementary material for: Discovery of Novel Multi-target Inhibitor of angiotensin type 1 receptor and neprilysin inhibitors from Traditional Chinese Medicine
Source: Sci Rep. 2019 Nov 7;9:16205. doi: 10.1038/s41598-019-52309-z (PMC6838339; doi:10.1038/s41598-019-52309-z)
Supplement: Supplementary file 1 — Table S1, Table S2, Table S3, Table S4, Table S5, Figure S1, Figure S2, Figure S3, Table S6, Table S7 [file 41598_2019_52309_MOESM1_ESM.docx]

**Discovery of Novel Multi-target Inhibitor of angiotensin type 1 receptor and neprilysin inhibitors from Traditional Chinese Medicine**

**Xiaoqian Huo, Liansheng Qiao, Yankun Chen, Xi Chen,** **Yusu He and Yanling Zhang***

School of Chinese Materia Medica, Beijing University of Chinese Medicine, Beijing, 100102, China

*corresponding. zhangyanling@bucm.edu.cn

Table S1 Fit values of compounds in testing set for optimal SBP model of AT1 agonists

| Compounds | Types | Fit value |
| --- | --- | --- |
| R794847 | AT1 agonist | 0.63 |
| R795100 | AT1 agonist | 0.53 |
| R801832 | AT1 agonist | 0.63 |
| R781253 | AT1 inverse agonist | 0.21 |
| R781254 | AT1 inverse agonist | 0.22 |
| R791212 | AT1 antagonist | 0.19 |

Table S2 Top 10 potential AT1 antagonists by combinatorial virtual screening

| ID | Structure | Name | Source plant | CDOCKER ENERGY | Fit value |
| --- | --- | --- | --- | --- | --- |
| 9185 |  | Gyrophoric acid | *Parmelia saxatilis* | 26.26 | 0.76 |
| 3017 |  | Calyxin H | *Alpinia pinnanensis .* | 26.82 | 0.76 |
| 21690 |  | 1,3,6-Tri(4-hydroxybenzyl)-4-methoxydihydrophenanthrene-2,7-diol | *Bletilla formosana* | 31.45 | 0.75 |
| 20314 |  | Stephaflavone A | *Stephania tetrandra* | 29.00 | 0.75 |
| 9644 |  | Hordatine A | *Hordeum vulgare* | 43.20 | 0.72 |
| 3020 |  | Calyxin K | *Alpinia blepharocalyx* | 38.61 | 0.73 |
| 15105 |  | Murramarin A | *Murraya exotica* | 30.34 | 0.76 |
| 9855 |  | 1ξ-Hydroxy-1,7-bis(4-hydroxy-3-methoxyphenyl)-6-heptene-3,5-dione | *Curcuma longa* | 40.88 | 0.74 |
| 17432 |  | Piperaduncin C | *Piper aduncum* | 37.38 | 0.75 |
| 3022 |  | Calyxin M | *Alpinia blepharocalyx* | 35.50 | 0.75 |

Table S3 Top 10 potential NEP inhibitors by combinatorial virtual screening

| ID | Structure | name | source plant | CDOCKER ENERGY | Fit value |
| --- | --- | --- | --- | --- | --- |
| 18527 |  | Ramalic acid | Usnea longissima. | 0.83 | 38.75 |
| 6186 |  | 3,3'-Diiodothyronine | Bos taurus domesticus | 0.64 | 68.22 |
| 16967 |  | Perlatolic acid | Cladonia stellaris | 0.74 | 49.88 |
| 21111 |  | (2S)-5,7,2',5'-Tetrahydroxyflavanone 7-O-β-D-glucuronopyranoside | Scutellaria amabilis | 0.81 | 43.21 |
| 19748 |  | Sennidin C | Rheum officinale | 0.76 | 48.91 |
| 3518 |  | Chicoric acid | Cichorium intybus | 0.61 | 82.71 |
| 5546 |  | Dihydrobaicalin | Scutellaria baicalensis | 0.87 | 39.29 |
| 13445 |  | 7-(6-O-Malonyl-β-D-glucopyransyloxy)-3-(4-hydroxyphenyl)-4H-1-benzopyran-4-one | Pueraria lobata | 0.70 | 64.65 |
| 21868 |  | 3,5,3'-Triiodothyronine | Bos taurus domesticus | 0.68 | 69.74 |
| 19850 |  | Shogasulfonic acid C | Zingiber officinale | 0.76 | 57.12 |

Table S4 Key interactive residues of AT1 antagonists based on docking computation

| Compounds | Hydrogen Bond | Hydrophobic | Electrostatic |
| --- | --- | --- | --- |
| ZD7155 | TYR35, ARG167 | VAL108, TRP84, ILE31, PRO285, ILE288, PHE77, TYR292, ALA163 |  |
| Valsartan | TYR35, ARG167, SER105 | ILE288, TRP84, ALA163, LEU112, PHE182, TRP253, VAL108 | ARG167 |
| Gyrophoric acid | THR88, ARG167, TYR184, LYS199, SER105, TRP84, PHE182 | TRP84, PHE182, TYR87, PRO162, VAL108 | ARG167 |

Table S5 Key interactive residues of NEP inhibitors based on docking computation

| Compounds | Hydrogen Bond | Hydrophobic | Electrostatic |
| --- | --- | --- | --- |
| Phosphoramidon | ARG110, TYR545, GLU584, HIS711, ARG717, GLU646, ASN542, SER546, HIS587 | VAL580, TRP693, HIS583 | HIS711, ZN^2+^, ARG717, MET579 |
| Sacubitril | ASN542, HIS711, ALA543 | TYR545, HIS587, PHE544, HIS711 | ZN^2+^ |
| 3,5,3'-triiodothyronine | GLU584, HIS711, ASN542, ALA543 | VAL710, HIS587, HIS711 | HIS711, ZN^2+^, GLU646 |

| 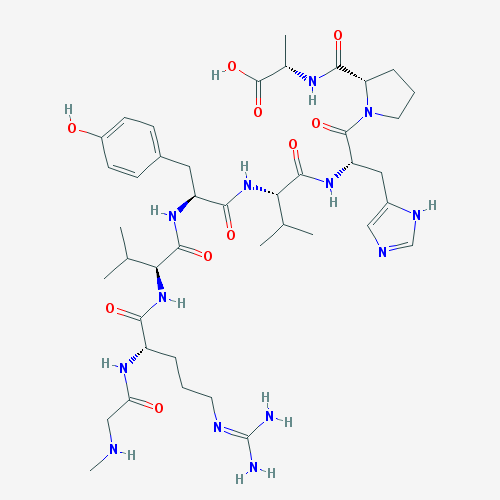 | 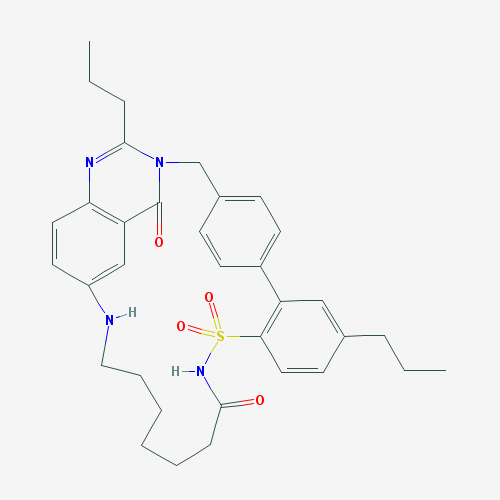 | 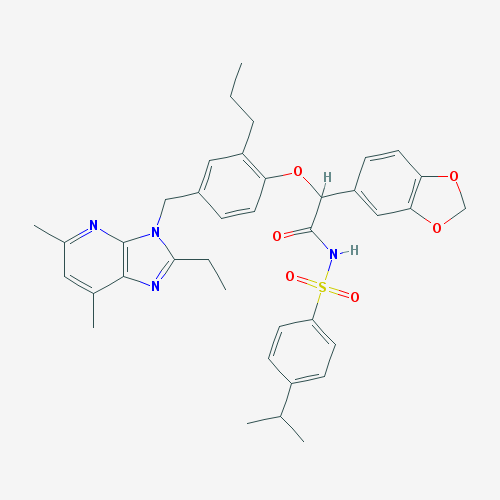 | 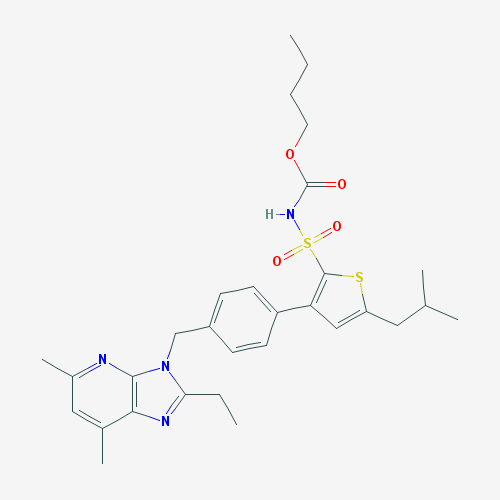 | 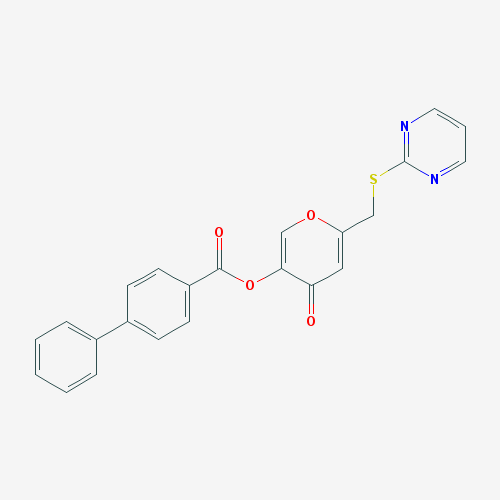 |
| --- | --- | --- | --- | --- |
| BDBM50009338 | BDBM50049193 | BDBM50117909 | BDBM50049189 | BDBM50393905 |
| IC_50_=0.57nM | IC_50_=4 nM | IC_50_=13 nM | IC_50_=1.1 nM | IC_50_=79,000 nM |
| 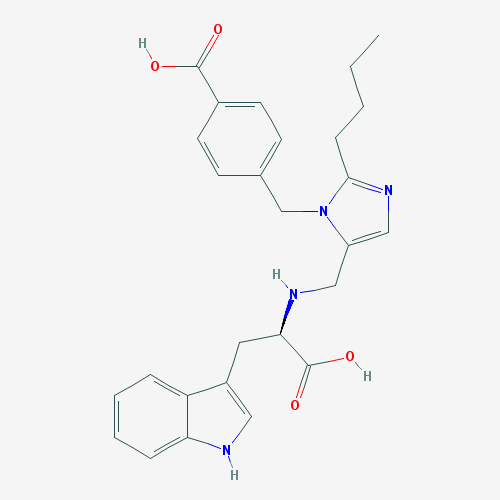 | 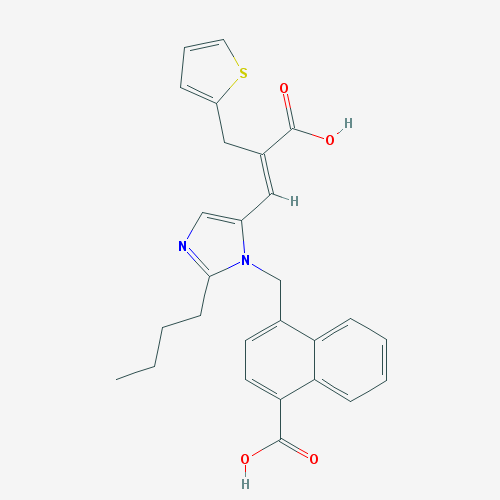 | 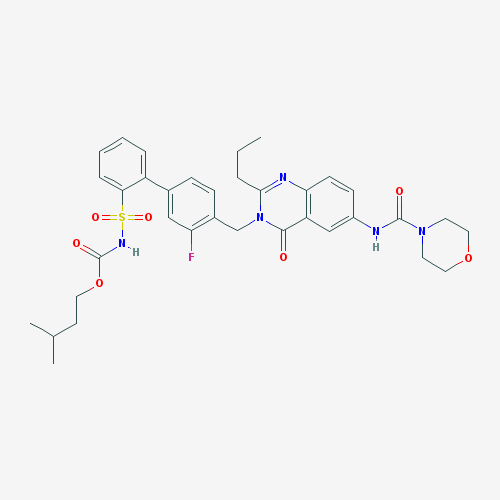 | 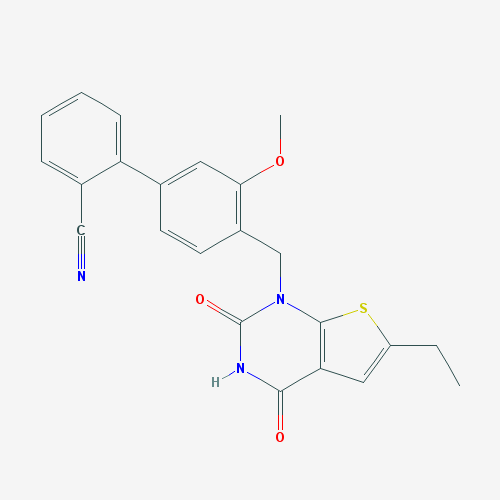 | 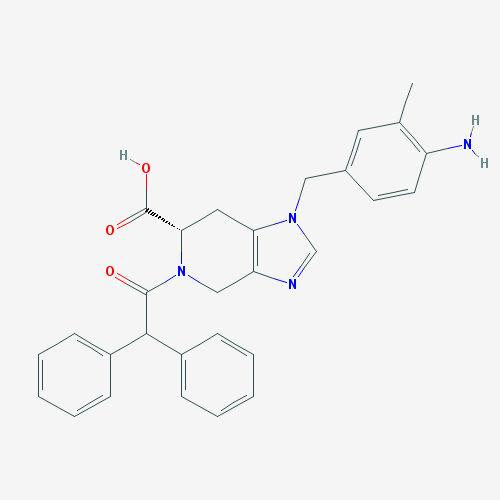 |
| BDBM50284297 | BDBM50282362 | BDBM50283317 | BDBM99976 | BDBM50010361 |
| IC_50_=2.9 nM | IC_50_=31 nM | IC_50_=0.71 nM | IC_50_=1.9 nM | IC_50_=100,000 nM |
| 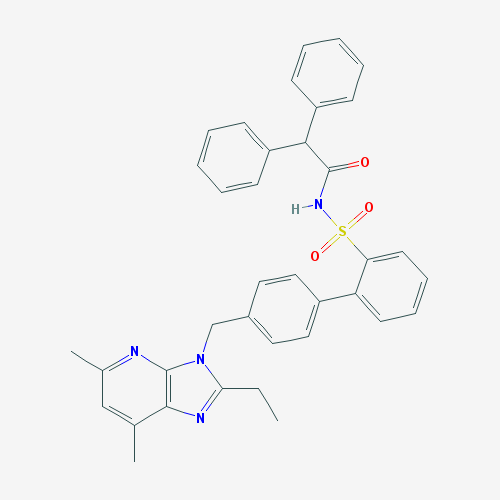 | 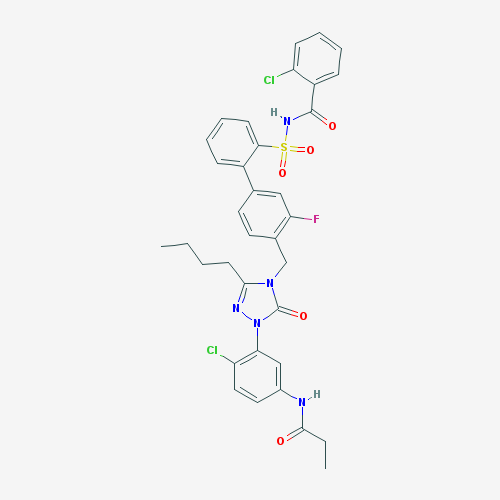 | 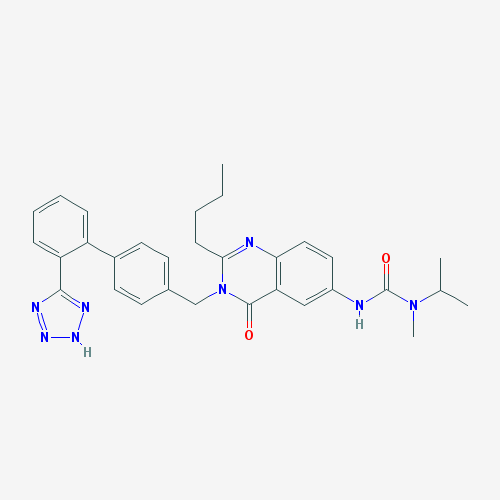 | 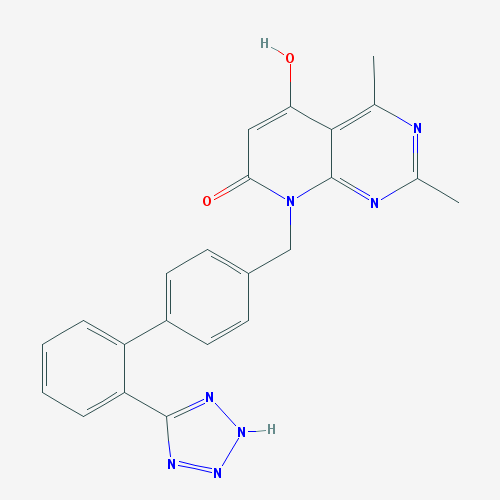 | 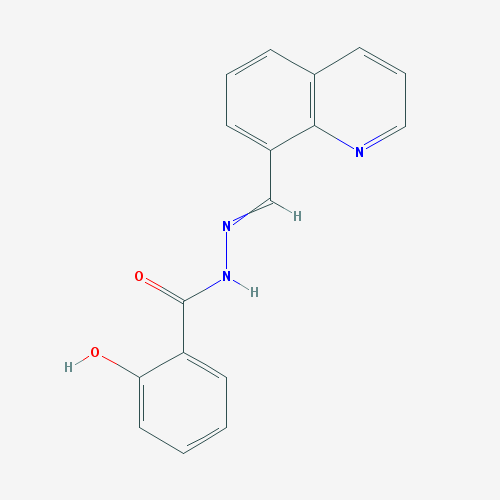 |
| BDBM50049199 | BDBM50030692 | BDBM50041969 | BDBM50067536 | BDBM54371 |
| IC_50_=0.05 nM | IC_50_=0.072 nM | IC_50_=0.1 nM | IC_50_=0.17 nM | IC_50_=1,559 nM |
| 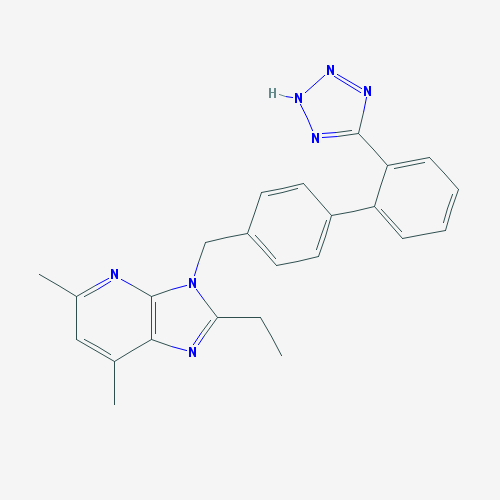 | 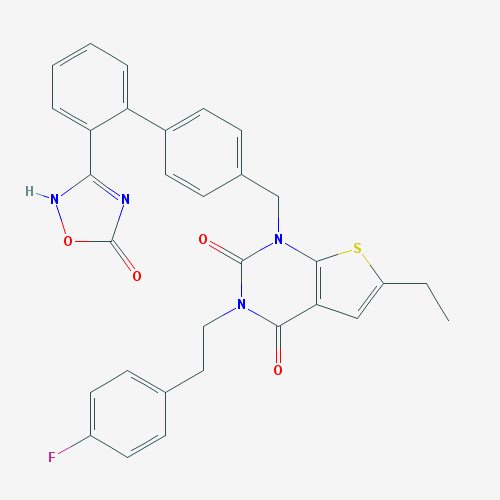 | 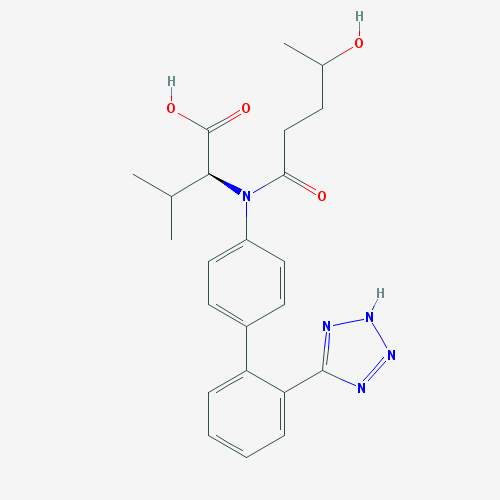 | 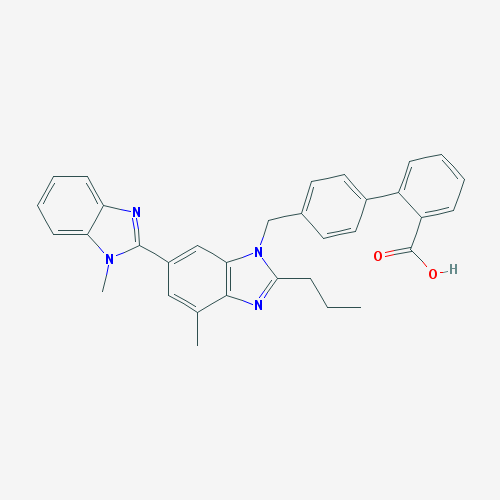 | 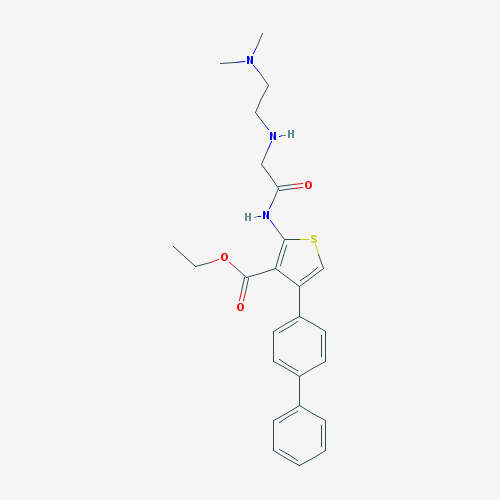 |
| BDBM50009718 | BDBM99943 | BDBM50128500 | BDBM50043280 | BDBM51918 |
| IC_50_=0.3 nM | IC_50_=0.34 nM | IC_50_=0.47 nM | IC_50_=0.49 nM | IC_50_=9,333 nM |

Figure S1 Training set of LBP modeling for AT1 antagonists

| 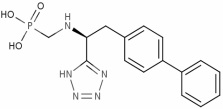 | 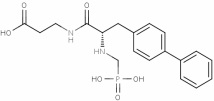 | 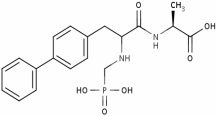 | 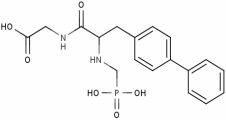 | 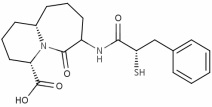 |
| --- | --- | --- | --- | --- |
| 290698  IC_50_=1nM | 285038  IC_50_=1.9nM | 146594  IC_50_=3nM | 357903  IC_50_=5nM | 325056  IC_50_=9nM |
| 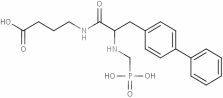 | 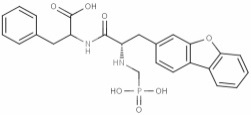 | 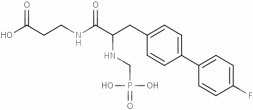 | 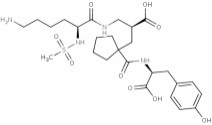 | 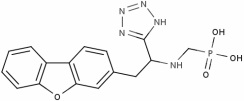 |
| 145937  IC_50_=11nM | 413724  IC_50_=12nM | 146631  IC_50_=14nM | 42583  IC_50_=20nM | 148056  IC_50_=114nM |
| 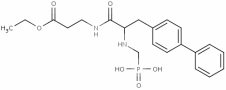 | 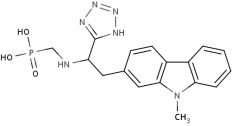 | 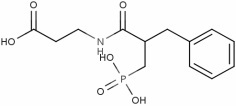 | 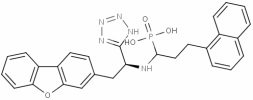 | 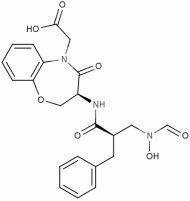 |
| 342768  IC_50_=120nM | 147041  IC_50_=140nM | 149131  IC_50_=207nM | 147964  IC_50_=210nM | 89760  IC_50_=290nM |
| 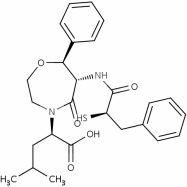 | 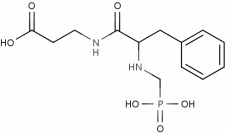 | 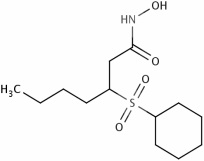 | 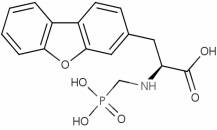 | 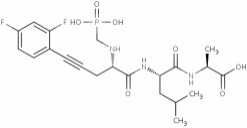 |
| 311838  IC_50_=330nM | 147400  IC_50_=1150nM | 77258  IC_50_=1650nM | 146936  IC_50_=2300nM | 147847  IC_50_=5800nM |
| 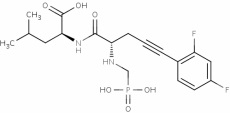 | 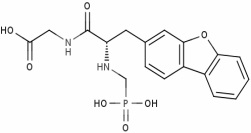 | 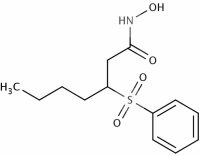 |  |  |
| 289150  IC_50_=6700nM | 147730  IC_50_=10000nM | 263501  IC_50_=18000nM |  |  |

Figure S2 Training set of LBP modeling for NEP inhibitors


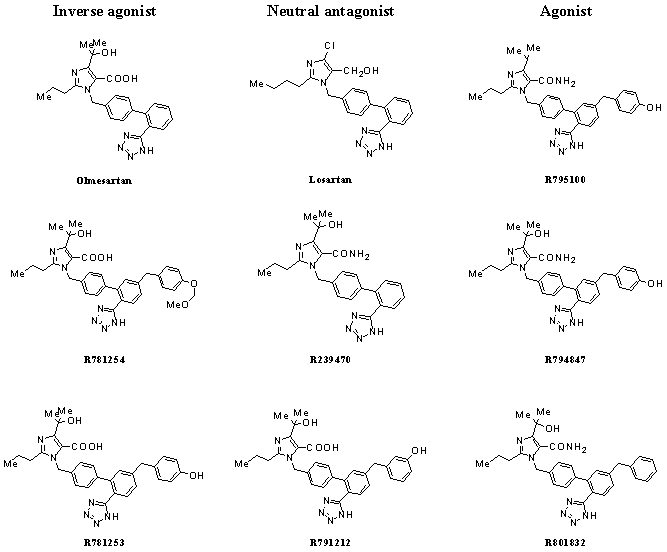


Figure S3 Testing set of SBP modeling for AT1 agonists

Table S6 The NEP inhibition activity of control group

| Group 1  0% hydrolyze | Group 2  100% hydrolyze | Group 3  with DL-Thiorphan | Inhibition ratio |
| --- | --- | --- | --- |
| 32.643 | 43.647 | 34.773 | 0.81 |
| 32.676 | 44.77 | 33.073 | 0.97 |
| 32.97 | 42.937 | 34.788 | 0.82 |

Table S7 The NEP activity assay of fluorescence

| 0% hydrolyze | 100% hydrolyze | Group 4  50nM | Group 5  100nM | Group 6  500nM |
| --- | --- | --- | --- | --- |
| 21.702 | 26.318 | 25.187 | 23.108 | 22.971 |
|  |  | 25.243 | 23.253 | 23.477 |
|  |  | 25.466 | 23.28 | 24.047 |
| 21.76 | 26.484 | 25.492 | 23.391  24.122  25.025 | 24.91 |
|  |  | 25.558 |  | 25.257 |
| 21.921 | 26.758 | 25.662  25.696  25.803 | 25.304  26.196  26.919 | 25.746 |
|  |  |  |  | 25.899 |
